# The relation between autonomy support and music enjoyment in online learning for music undergraduates in the post-COVID-19 era

**DOI:** 10.3389/fpsyg.2022.1062546

**Published:** 2022-12-08

**Authors:** Yan-Han Zhang, Yue-Han Zhao, Yuan-Yu Luo, Xiantong Yang, Dawei Tan

**Affiliations:** ^1^College of Music, Hebei Normal University, Shijiazhuang, Hebei, China; ^2^Department of Cultural and Creative Arts, The Education University of Hong Kong, Tai Po, Hong Kong SAR, China; ^3^Faculty of Education, University of Cambridge, Cambridge, England, United Kingdom; ^4^College of Music, Ningbo University, Ningbo, Zhejiang, China; ^5^Faculty of Psychology, Beijing Normal University, Beijing, China; ^6^College of Teacher Education, Zhejiang Normal University, Jinhua, Zhejiang, China; ^7^Department of Music, Taiyuan Normal University, Taiyuan, Shanxi, China

**Keywords:** music major undergraduates, online learning, autonomy support, music enjoyment, post-pandemic era

## Abstract

Music enjoyment is considered to predict music-related academic performance and career choice. Although relevant research in non-music fields has demonstrated the association between teachers’ autonomy support and students’ academic enjoyment, it remains unknown whether this association is valid in the music discipline. In addition, in the post-COVID-19 era, online education has become a common way of teaching and learning for music undergraduates. In the form of online learning, the mechanisms mediating teachers’ music autonomy support and students’ music academic enjoyment are also unknown. This study draws on Pekrun’s theory of achievement emotions and control values to explore the mediating role of attributions and values in the association between autonomous support and academic achievement. In this study, 270 undergraduates majoring in music eventually completed the online surveys. Results from structural equation modeling indicated that autonomy support positively predicted music enjoyment and that attributions (i.e., internal attribution and external attribution) and values (i.e., intrinsic value, attainment value, utility value) mediated the association between autonomy support and music enjoyment. The findings also provide insights into possible avenue for promoting music enjoyment emotion during online teaching in the post-COVID-19 era. Implications and limitations are discussed in the study.

## Introduction

Since the outbreak of the COVID-19 pandemic, online education has become a common way of teaching and learning in higher education institution worldwide (Cucinotta and Vanelli, 2020). With the advent of the post-COVID-19 era, although the offline education mode is gradually recovering, online education is still one of the common modes of teaching and learning in Chinese universities (Ma et al., 2022; Syam and Achmad, 2022). However, unlike offline education where teachers and students can interact directly face-to-face, online education is characterized by the difficulty of physical and emotional interaction between teachers and students, which leads to problems such as limited student engagement and reduced interest in learning (Tsegay et al., 2022). Studies have found that in the higher education institutions, students’ online academic engagement is significantly associated with their academic achievement (Omar et al., 2021), while achievement emotions have a stable positive effect on promoting students’ online academic engagement (Deng et al., 2022). Hence, achievement emotions are thought to enhance students’ online academic engagement and in turn benefit their learning outcomes (Leino et al., 2021). However, studies have shown that students who take online courses have fewer achievement emotions than those who take offline courses (Stephan et al., 2019). Therefore, it is important to promote students’ achievement emotions during online education.

The control-value theory of achievement emotions suggests that characteristics of the learning environment are indirect predictors of students’ emotions (Pekrun, 2006). Enjoyment as one of the achievement emotions is considered to be beneficial in enhancing students’ interest, self-regulatory behaviors, deep learning strategies, and sense of accomplishment (Pekrun, 2006; Goetz et al., 2014). Teachers’ autonomy support, as a component of the social environment, is one of the most important social antecedents of enjoyment emotions (Pekrun, 2006). As a component of cognitive appraisal, control appraisal and value appraisal are thought to operate as a mediator between the learning environment (e.g., autonomy support) and the achievement emotion (e.g., enjoyment) (Pekrun, 2006; Sorić et al., 2013; Goetz et al., 2014). Further, control attributions are in turn considered to be related to internal and external attributions (Pekrun et al., 2002; Pekrun, 2006; Pekrun and Perry, 2014). Thus, autonomy support is thought to further predict subsequent enjoyment emotions by mediating cognitive evaluations of attribution and value (Pekrun, 2006).

In music education, the aesthetic experience that music has produces aesthetic emotions which include enjoyment (Brattico et al., 2013). Enjoyment is therefore considered to be one of the important emotions in music education (Lee, 2009). Meanwhile, autonomy support has been recognized and used by music teachers for its potential to promote music learning (Evans, 2015). Although studies have demonstrated that teacher autonomy support in music education has a positive correlation with student enjoyment at the secondary level (Papageorgi and Economidou Stavrou, 2021), there is limited research on the association between teachers’ autonomy support and students’ music enjoyment in the form of online music education and also at the higher education level (Bonneville-Roussy et al., 2013). Therefore, this study will use the control-value theory of achievement emotions as a theoretical framework to validate the relationship between autonomy support and music enjoyment, and the mediating role of attribution and value, to understand the antecedents and consequences of music majors’ enjoyment of music in online education and its mechanisms of action.

### Autonomy support and music enjoyment

Teacher autonomy support is one of the components of the teaching and learning environment (Pekrun, 2019a; Raccanello et al., 2022). It refers to students’ perceived teachers’ supportive behaviors towards students and includes all supportive behaviors of teachers that promote students’ personal and academic development, describing the extent to which students perceive that teachers help and feel that teachers care about their learning process (Monteiro et al., 2017). More specifically, autonomy support is an instructional practice strategy that fosters student motivation by promoting a sense of self-acknowledged learning, by giving students meaningful choices, matching course content to students’ interests and ability levels, providing students with explanations and justifications for their learning, and using non-controlled instructional language (Jang et al., 2016).

Students could experience a range of different achievement emotions, which are also referred to as emotional arousal directly related to achievement activities or achievement outcomes (Pekrun and Perry, 2014). Enjoyment, as one of them, is a positively high arousal emotion (Pekrun, 2006). Additionally, achievement emotions are key predictors of students’ academic performance and career choices (Pekrun, 2019a) and are shaped by environmental, situational and personal factors of the school experience (Pekrun, 2006; Pekrun et al., 2017). Pekrun (2019a) categorized achievement emotions into three dimensions: (a) valence, which indicates whether the emotion is positive (e.g., pride) or negative (e.g., anger); (b) the degree of activation, which is the degree of physiological arousal and can be divided into activating emotions (e.g., joy) and deactivating emotions (e.g., sadness); (c) object focus, that is, whether the emotion is described as activity-related (e.g., enjoyment, anger) or outcome-related (e.g., pride, anxiety). Within the different categories of emotions, the enjoyment belongs to activity emotions, which refer to emotions arising from achievement-related activities. It is also a positive and activating emotion that contributes to problem-solving approach-oriented behaviors (Fredrickson, 2001; Pekrun, 2006). It has been shown to have an important role in promoting students’ interests, self-regulatory behaviors, deep learning strategies and achievement (Pekrun, 2006; Goetz et al., 2014).

The control-value theory of achievement emotions suggests that shaping the educational environment in an appropriate way can help change achievement emotions (Pekrun, 2006). As discussed earlier, autonomy support is one of the elements of the educational environment (Pekrun, 2006). Emotions are also considered to be related to the environment (Pekrun, 2006; Pekrun and Perry, 2014). Therefore, autonomy support is likely to be associated with emotions. In the case of enjoyment, research has shown a positive association between autonomy support and positive emotions (Barrable, 2020; Wang and Hu, 2022). Enjoyment, as a positive emotion, has also been shown to be positively associated with autonomy support (Simonton et al., 2021; Zimmermann et al., 2021).

For music education, the aesthetic experiences that occur in music education and enjoyment generate aesthetic emotions (Brattico et al., 2013). One of these aesthetic emotions is enjoyment (Brattico et al., 2013). Therefore, enjoyment is an important emotion in music education (Lee, 2009). Autonomy support also has many benefits for music education. For example, autonomy support is considered to promote students’ intrinsic motivation for music learning and autonomy support from the music teacher is associated with students’ harmonious enthusiasm for music learning (Bonneville-Roussy et al., 2013; Evans, 2015). Furthermore, researchers found that autonomy support and well-being among UK music undergraduates showed a positive association (Bonneville-Roussy et al., 2020). According to the PERMA framework, positive emotion is the element of well-being (Seligman, 2012, 2018), while enjoyment is one of the positive emotions (Pekrun, 2006). Thus, the autonomy support might positively predict music enjoyment. In fact, studies have focused on the association between autonomy support and enjoyment in music education. A study on music classroom education in secondary schools found a positive association between teachers’ autonomy support and students’ music enjoyment levels (Papageorgi and Economidou Stavrou, 2021). However, for music education, there is limited attention has been paid on the association between teachers’ autonomy support and students’ music enjoyment emotions in higher education and online education environment. In light of the above discussion, this study assumes that online learning in the field of music is in line with the general field of learning. Based on this, this study proposes the hypothesis that autonomy support might positively predict music enjoyment (H1).

### Attribution as the potential mediation of autonomy support and music enjoyment

As discussed earlier, autonomy support is related to the educational environment, and enjoyment is one of the achievement emotions (Goetz et al., 2014; Pekrun, 2019a). Cognitive appraisal is considered the mediating factor between the learning environment and achievement emotions (Sorić et al., 2013). According to the control-value theory of achievement emotions, cognitive appraisal consists of control appraisals (Pekrun, 2006).

Control beliefs arise from individuals’ subjective estimates of the extent to which they influence and predict outcomes and events throughout the lifespan (Chipperfield et al., 2016). It includes students’ perceived control over behaviors and outcomes and is associated with personal assessment of competence, expected outcomes and success/failure attributions (Pekrun et al., 2002; Pekrun, 2006; Pekrun and Perry, 2014; Pekrun, 2019a). High control beliefs increase students’ ability to perform and expectations of success (Pekrun et al., 2002; Pekrun, 2006), which is also associated with achievement, effort, intrinsic motivation and self-monitoring behavior in university students (Perry et al., 2001). Attribution of outcomes is a retrospective evaluation of the causes of success and failure (Pekrun, 2006). Success attributions are divided into internal reasons (i.e., reasons internal to the individual, e.g., ability, effort) and external reasons (i.e., reasons due to external factors, e.g., task difficulty, luck). Therefore, the control attributions in this study’s hypothesis are divided into internal and external attributions.

Low control beliefs attribute success or failure to external sources or imply a lack of ability to produce the desired action (Perry et al., 2001; Pekrun, 2006, 2019a). It is assumed that if teachers attempt to allow students to make important learning decisions on their own, their belief appraisals will improve accordingly (Pekrun, 2006; Linnenbrink-Garcia et al., 2016). Research has shown that adopting an autonomy supportive teaching approach could facilitate the development of internal control beliefs (Pekrun and Perry, 2014). Furthermore, the research found that autonomy support positively predicts attributions (Pekrun, 2019a). Therefore, this study hypothesizes that autonomy support might negatively predict external attributions (H2-1) and positively predict internal attributions (H2-2), separately.

Control appraisals are predictors of achievement emotions (Pekrun et al., 2017; Simonton and Garn, 2020). In general, high levels of control beliefs predicted higher levels of positive emotions (e.g., enjoyment, pride) and lower levels of negative emotions (e.g., despair, anxiety; Elliot and Pekrun, 2007; Goetz et al., 2014). Enjoyment is positively associated with higher levels of perceived control (Goetz et al., 2010; Pekrun et al., 2011; Bailey et al., 2014; Hagenauer and Hascher, 2014). Therefore, this study hypothesizes that external attributions might negatively predict music enjoyment (H2-3) and internal attributions might positively predict music enjoyment (H2-4). To sum up, this study proposes the hypothesis that attribution might play a mediating role between autonomy support and music enjoyment (H2).

### Value as the potential mediation of autonomy support and music enjoyment

In the same vein as control appraisal, value appraisal, as part of cognitive appraisal, is considered to be a mediating factor between the learning environment (e.g., autonomy support) and the emotion of achievement (e.g., enjoyment; Pekrun, 2006; Sorić et al., 2013; Goetz et al., 2014). Value appraisal refers to the value and interest that individuals place on tasks and activities (Pekrun, 2019b; Wigfield and Eccles, 2020), including three types: attainment value, intrinsic value and utility value. Student control (Pekrun, 2019b; Wigfield and Eccles, 2020) and value appraisal are seen as mediators of the association between characteristics of the learning environment and different achievement emotion experiences (Pekrun and Perry, 2014). Attributions and values are two components of appraisal in the control value theory of achievement emotions, as well as mediators of environment and Emotion (Pekrun and Stephens, 2010). In the hypothesis of this study, the value appraisal is divided into three categories: achievement, intrinsic and utility value.

Beliefs of autonomy support are related to students’ value status (PSimonton et al., 2021). The relationship between autonomy support and value appraisals has been examined in other educational settings. Studies have found that autonomy-supportive teaching increases students’ evaluations of self-efficacy and intrinsic value (Buhr et al., 2019; Ekatushabe et al., 2021; Klee et al., 2022) and also positively predicts students’ intrinsic value (Zimmermann et al., 2021). Thus, music students’ perceived teacher online autonomy support can potentially be a predictor of value (Pekrun, 2006; Ng et al., 2016). Hence, this study hypothesizes that autonomy support might positively predict achievement value (H3-1), intrinsic value (H3-2) and utility value (H3-3).

Value appraisals are predictors of achievement emotions (Pekrun et al., 2017; Simonton and Garn, 2020). In general, high levels of value beliefs predicted higher levels of positive emotions (e.g., enjoyment, pride) and lower levels of negative emotions (e.g., despair, anxiety; Elliot and Pekrun, 2007; Forsblom et al., 2022). Enjoyment is positively associated with positive valuation (Goetz et al., 2010; Pekrun et al., 2011; Bailey et al., 2014; Hagenauer and Hascher, 2014). Some studies have shown a positive relationship between utility value and enjoyment (Putwain et al., 2021), as rewards and success can be seen as personal values. Students could experience enjoyment in the classroom and still be driven by utility values (Frenzel et al., 2007b). Higher values beliefs are strongly associated with positive emotions such as enjoyment (Pekrun, 2006, 2019b; Frenzel et al., 2007a). In a sample of high school students, control and value appraisals for physical education (PE) were positive predictors of enjoyment and negative predictors of boredom (Simonton1 et al., 2017; Zimmermann et al., 2021). Currently, limited research has focused on the mediating role of control and value on autonomy support as well as music enjoyment in music learning. Based on the existing studies, the present study hypothesizes that attribution and value are mediating variables for autonomy support and music enjoyment. Thus, the present study hypothesizes that achievement value (H3-4), intrinsic value (H3-5) and utility value (H3-6) might positively predict music enjoyment. In summary, this study proposes the hypothesis that value might play a mediating role between autonomy support and music enjoyment (H3).

### The present study

The above studies discussed the relationship between autonomy support, enjoyment and control, and value in both music and non-musical domains. Based on Pekrun’s (2006, 2019a) control-value theory of achievement emotions focusing on the antecedents and correlates of achievement emotions (Wang et al., 2017; Liu et al., 2018; Muwonge et al., 2018), we argue that the music domain’s variables conform to the psychological patterns of the general domain. Based on the discussion above, this study hypothesized that there might be a positive predictive association between autonomy support and music enjoyment, and attribution and value might play a mediating role between autonomy support and music enjoyment. The hypothesis of this study are as follows:

*H2-1*: Autonomy support negatively predicts external attribution.

*H2-2*: Autonomy support positively predicts internal attribution.

*H2-3*: External attribution negatively predicts music enjoyment.

*H2-4*: Internal attribution positively predicts music enjoyment.

*H3-1*: Autonomy support positively predicts attainment value.

*H3-2*: Autonomy support positively predicts intrinsic value.

*H3-3*: Autonomy support positively predicts utility value.

*H3-4*: Attainment value positively predicts music enjoyment.

*H3-5*: Intrinsic value positively predicts music enjoyment.

*H3-6*: Utility value positively predicts music enjoyment.

**Figure 1:**
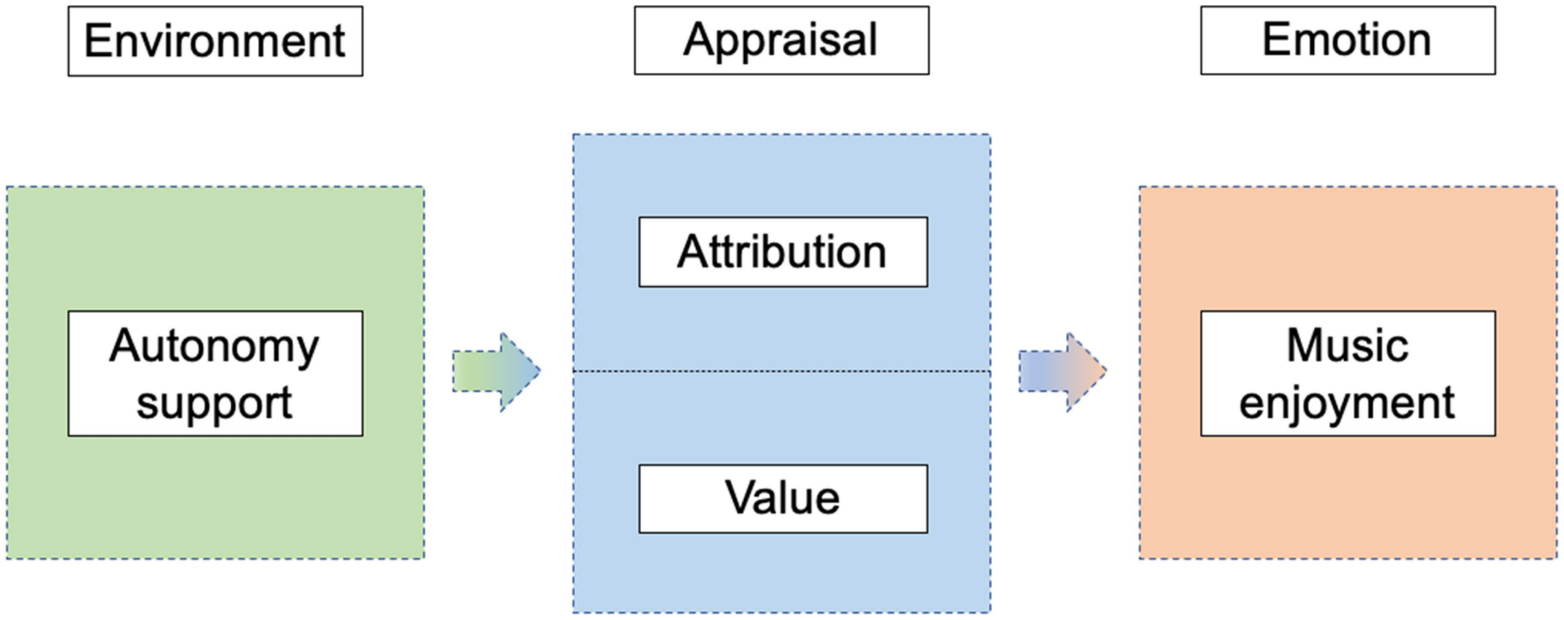
Mediation model of attributions between autonomy support and music enjoyment.

Taken together, this study proposes the following hypothetical model of the association between autonomy support and music enjoyment (Figure 1):

*H1*: Autonomy support is positively associated with music enjoyment.

*H2*: Attribution is the mediation of autonomy support and music enjoyment.

*H3*: Value is the mediation of autonomy support and music enjoyment.

## Materials and methods

### Participants

This study conducted data collection by means of an online questionnaire from 300 university students majoring in music at a university in the middle of China in September 2022, by using a whole group sampling (Cluster sampling) method. All participants participated voluntarily and were clearly informed of the purpose of the study, its significance, how the data would be kept confidential and what the data would be used for before participating in the study. There would be no rewards for participants. After removing incomplete or duplicate responses, 270 valid responses were used for data analysis. It included 71 male (26.3%) and 199 female (73.7%) students who were in their second (*N* = 116, 43%), third (*N* = 70, 25.9%) and fourth (*N* = 84, 31.1%) years of the music major.

### Measures

The questionnaire items were adapted from previous theories or researchers. In order to verify the accuracy of the questionnaire and to ensure its face validity, a forward-backward approach was used to professionally translate the original items into Chinese.

Although web-based questionnaire has some advantages such as less cost and less errors of data entry and coding, more rapidly return etc. (Van Gelder et al., 2010), there remains disadvantages that hamper the data collection (e.g., answering carelessly). Thus, optimizing the questionnaire design is important to enhance the reliability and validity of the data from web-based data questionnaires. In present study, we strictly controlled the survey length by decreasing item numbers and improve the and survey quality by setting careless detection items separately. In this way, the reliability of the web-based questionnaire has been guaranteed. The detailed results of reliability are as follows.

### Questionnaire of autonomy support

The autonomy support questionnaire (Assor et al., 2002) was used to measure autonomy support for students during online learning, with a total of 13 items divided into three dimensions, including providing choice (e.g., In music class, the teacher encouraged us to try to solve problems on our own), critique and reflection (e.g., The music teacher showed me how to solve problems on my own) and promoting understanding (e.g., The music teacher explained why it was important to learn some subjects). Participants scored on a 5-point Likert scale (1 = *never*, 5 = *always*), with higher scores indicating higher teacher AU for students. The model fit indices in this study showed good construct validity (*χ*^2^/df = 17.277/7, CFI = 0.972, TLI = 0.939, RMSEA = 0.074, SRMR = 0.028), Cronbach’s *α* = 0.759.

### Questionnaire of external attribution

The external attribution questionnaire (Lefcourt et al., 1979) was used to measure students’ external attributions of success in online teaching, with a total of six items. It was divided into two dimensions, including difficulty (e.g., I got high marks in music lessons a few times because the teacher scored too loosely) and luck (e.g., Success in music exams sometimes depends on a little bit of luck). Participants scored on a 5-point Likert scale (1 = *completely disagree*, 5 = *completely agree*), with higher scores indicating higher external attributions of success. The model fit indices in this study showed good construct validity (*χ*^2^/df = 17.277/7, CFI = 0.972, TLI = 0.939, RMSEA = 0.074, SRMR = 0.028), Cronbach’s α = 0.759.

### Questionnaire of internal attribution

The internal attribution questionnaire (Lefcourt et al., 1979) was used to measure students’ internal attributions of success in online learning, with a total of six items divided into two dimensions, including the ability (e.g., the most important factor in getting a good grade is my academic ability in music) and effort (e.g., whenever I get a good grade, it is because I have studied hard in the subject). (e.g., whenever I get a good grade, it is because I have studied hard in the subject). Participants scored on a 5-point Likert scale (1 = *completely disagree*, 5 = *completely agree*), with higher scores indicating higher internal attributions of success and the model fit indices indicating good construct validity (*χ*^2^/df = 9.520/7, CFI = 0.993, TLI = 0.985, RMSEA = 0.037, SRMR = 0.023), Cronbach’s *α* = 0.757.

### Questionnaire of values

The values questionnaire (Eccles and Wigfield, 1995) was used to measure students’ perceptions of value in online teaching, with a total of nine items divided into three dimensions: attainment value, intrinsic value and utility value, include attainment value (e.g., I like what I learn in music lessons), intrinsic value (e.g., I do need more experience and I think about it a lot so I learn more) and utility value (e.g., I think it is important to understand what I learn in music lessons). Participants scored on a 5-point Likert scale (1 = *completely disagree*, 5 = *completely agree*), with higher scores indicating higher agreement with the value and model fit indices indicating good construct validity (*χ*^2^/df = 57.222/22, CFI = 0.968, TLI = 0.947, RMSEA = 0.077, SRMR = 0.036), and good reliability (Cronbach’s *α* = 0.876).

### Questionnaire of music enjoyment

The music enjoyment questionnaire (Pekrun et al., 2011) was used to measure students’ level of music enjoyment with a total of 3 items (e.g., I enjoy taking music classes). Participants rated their enjoyment on a 5-point Likert scale (1 = *completely disagree*, 5 = *completely agree*), with higher scores indicating higher levels of music enjoyment.

Convergent validity is an important part of construct validity which is assessed by averaging variance extracted (AVE). Hair et al. (2011) suggest that the AVE value should be higher than 0.50. In the present study, the AVE value of the autonomy support questionnaire and the internal attribution questionnaire are 0.534 and 0.644 separately, meaning these constructs has adoptable convergent validity.

### Data analysis

Firstly, the mean, standard deviation, Kendall and Pearson correlation coefficients were described using SPSS version numbers. The hypothesized multiple mediation model (MCM) was tested by PROCESS macro (Bolin, 2014).^1^ Additionally, a bias-corrected bootstrapping method was used to further examine the significance of mediating and moderating effects, whose 95% confidence interval did not contain zero indicating a salient effect (Bolin, 2014).

## Results

### Common method bias test

Harman single factor test was conducted in the present study. Based on the standard of characteristic root greater than one, nine factors in the present results were extracted from unrotated factor analysis. Furthermore, the variation of maximum factor variance interpretation was 32.23%, less than the critical standard of 40% (Zhou and Long, 2004), so no serious common method bias remained.

### Preliminary analysis

The results of the mean, standard deviation and correlation analyses are presented in Table 1 and show that among the demographic (age and grade) variables, only grade had a significant negative correlation (Negative correlation) for Autonomy support. Except for the demographic variables, all variables showed significant correlations as expected: (1) external attribution showed the expected negative correlations with the other variables (|*r*| between 0.206 and 0.449, *p* < 0.01); (2) while the non-demographic variables other than external attribution showed significant positive correlations (|*r*|between 0.299–0.670, *p* < 0.01).

**Table 1:** Means, standard deviations, and correlations among the main variables.

Variables	*M*	*SD*	Autonomy support	External attribution	Internal attribution	Attainment value	Intrinsic value	Utility value	Enjoyment	gender	grade
Autonomy support	3.93	0.65	1								
External attribution	2.62	0.59	−0.290^***^	1							
Internal attribution	3.56	0.59	0.299^***^	−0.206^**^	1						
Attainment value	4.03	0.59	0.609^***^	−0.449^***^	0.343^***^	1					
Intrinsic value	3.89	0.57	0.512^***^	−0.317^***^	0.403^***^	0.589^***^	1				
Utility value	4.26	0.59	0.482^***^	−0.380^***^	0.348^***^	0.670^***^	0.543^***^	1			
Enjoyment	3.99	0.67	0.548^***^	−0.413^***^	0.324^***^	0.647^***^	0.539^**^	0.561^**^	1		
Gender	–	–	−0.076	−0.111	0.026	−0.023	0.019	0.106	0.013	1	
Grades	–	–	−0.189^**^	0.034	0.046	−0.064	−0.029	0.028	−0.067	0.114	1

^**^p < 0.01, ^***^p < 0.001.

### Testing for the hypothetical model

Firstly, a direct model between autonomy support and music enjoyment was used to test hypothesis 1. The results indicated that autonomy support positively predicted music enjoyment (β = 0.560, *p* < 0.001), and the findings supported hypothesis 1.

**Figure 2:**
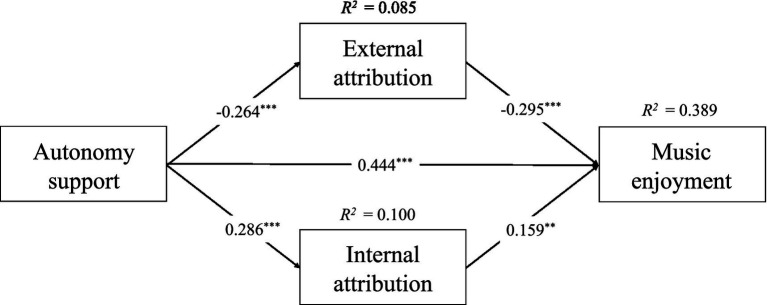
Mediation model of attributions between autonomy support and music enjoyment.

Secondly, a mediation model mediated by success attribution was constructed using the PROCESS macro (model No. 4), with success attribution divided into two dimensions, external attribution and internal attribution (Figure 2). As shown in Table 2, autonomy support had a significant negative predictive effect on external attribution (*β* = −0.264, *p* < 0.001), while external attribution also had a significant negative predictive effect on music enjoyment (*β* = −0.295, *p* < 0.001). In contrast, autonomy support had a significant positive predictive effect on internal attribution (*β* = 0.286, *p* < 0.001), while internal attribution also had a positive predictive effect on music enjoyment (*β* = 0.159, *p* < 0.001). The grade was not associated with any of these variables. In addition, the 95% confidence interval for external attribution was from 0.031 to 0.135 (not including zero) and the 95% confidence interval for internal attribution was from 0.011 to 0.093 (not including zero). The above results suggest that external attribution and internal attribution play a mediating role in the association between autonomy support and music enjoyment, and that research hypothesis 2 is supported.

**Table 2:** Mediation model of attributions between autonomy support and music enjoyment.

Predictors	External attribution			Internal attribution			Music enjoyment		
	*β*	*SE*	95% CI	*β*	*SE*	95% CI	*β*	*SE*	95% CI
Autonomy support	−0.264^***^	0.054	[−0.369, −0.158]	0.286^***^	0.053	[0.182, 0.390]	0.444^***^	0.054	[0.337, 0.550]
External attribution							−0.295^***^	0.058	[−0.408, −0.181]
Internal attribution							0.159^**^	0.058	[0.044, 0.273]
Grades	−0.015	0.041	[−0.095,0.066]	0.727	0.040	[−0.007, 0.152]	0.014	0.038	[−0.062, 0.090]
*R^2^*	0.085			0.100			0.389		
*F*	12.321			14.880			42.170		

Bootstrap sample size = 5,000; ^**^p < 0.01, ^***^p < 0.001.

**Figure 3:**
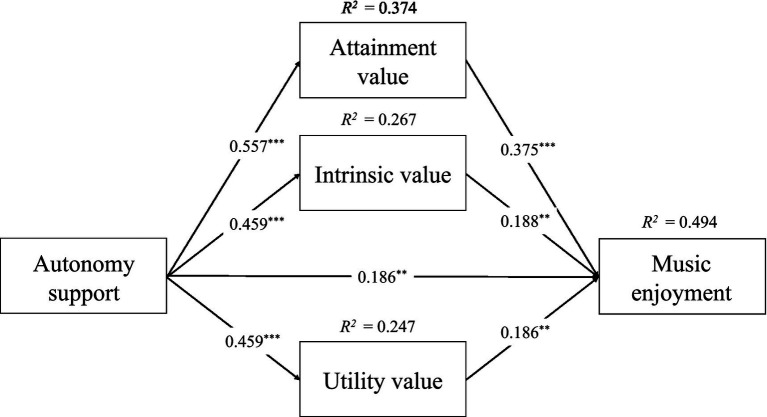
Mediation model of music values between autonomy support and music enjoyment.

Finally, the mediation model was constructed using the same PROCESS macro (model No. 4) with value as the mediator, with value divided into three dimensions: attainment value, intrinsic value and utility value (Figure 3). As shown in Table 3, autonomy support had a significant positive predictive effect on attainment value (*β* = 0.557, *p* < 0.001), while attainment value also had a significant positive predictive effect on music enjoyment (*β* = 0.357, *p* < 0.001). Autonomy support also showed a significant positive predictive effect on Intrinsic value (*β* = 0.459, *p* < 0.001), while Intrinsic value also had a positive predictive effect on music enjoyment (*β* = 0.188, *p* < 0.01). Besides, autonomy support had a significant positive predictive effect on utility value (*β* = 0.459, *p* < 0.001), while utility value also had a positive predictive effect on music enjoyment (*β* = 0.186, *p* < 0.01). The grade was not associated with attainment value, intrinsic value, and music enjoyment, while had a positive predictive effect on utility value (β = 0.086, p < 0.05). In addition, the 95% confidence interval for attainment value was 0.098–0.317 (not including zero), the 95% confidence interval for intrinsic value was 0.023–0.158 (not including zero), and the 95% confidence interval for utility value was 0.014–0.167 (not including zero). These results suggest that attainment value, intrinsic value and utility value play a mediating role in the association between autonomy support and music enjoyment, and that research hypothesis 3 is supported.

**Table 3:** Mediation model of music values between autonomy support and music enjoyment.

Predictors	Attainment value			Intrinsic value			Utility value			Music enjoyment		
	*β*	*SE*	95% CI	*β*	*SE*	95% CI	*β*	*SE*	95% CI	*β*	*SE*	95% CI
Autonomy support	0.557^***^	0.044	[0.450, 0.644]	0.459^***^	0.047	[0.368, 0.551]	0.459^***^	0.049	[0.362, 0.555]	0.186^**^	0.060	[0.069, 0.304]
Attainment value										0.375^***^	0.077	[0.224, 0.527]
Intrinsic value										0.188^**^	0.067	[0.056, 0.320]
Utility value										0.186^**^	0.069	[0.050, 0.322]
Grades	0.037	0.0339	[−0.030, 0.104]	0.047	0.036	[−0.023, 0.117]	0.086^*^	0.038	[0.012, 0.160]	−0.009	0.035	[−0.078, 0.060]
*R^2^*	0.374			0.267			0.247			0.494		
*F*	79.797			48.614			43.893			51.527		

Bootstrap sample size = 5,000; ^*^p < 0.05, ^**^p < 0.01, ^***^p < 0.001.

## Discussion

This study focuses on the association between autonomy support and music enjoyment in the online learning of music major undergraduates in the post-COVID-19 era. Based on Pekrun’s (2006) control-value theory of achievement emotions, the study clarifies the positive predictive association of autonomy support on music enjoyment and the mediating role of attribution and value. The results support the whole hypothesis of this study. In summary, the findings of this study contribute to understanding the positive outcomes of autonomy support and provide insights into ideas for enhancing students’ music enjoyment.

### Autonomy support is positively associated with music enjoyment

The results show that autonomy support is positively associated with music enjoyment. That is, when students receive higher levels of autonomy support, music enjoyment is also higher. This also supports the control-value theory of achievement emotions, which is consistent with previous research that autonomy support environments foster control and value beliefs that ultimately promote positive academic emotions (e.g., enjoyment; Simonton et al., 2021). In terms of teaching strategies, teachers’ autonomy support conveys supportive interpersonal messages and attempts to understand from the students’ perspective (Reeve, 2015). They offer students choices, make them feel understood and allow for critique. There was a significant indirect correlation between teachers’ autonomy support and students’ emotional responses to learning (Wang et al., 2017). In this study, the focus was on the music discipline. The results of this study provide evidence to support the cross-cultural application of the control-value theory of achievement emotions hypothesis, and it highlights the importance of promoting learners’ academic emotions through teachers’ autonomy support.

### The mediating role of attribution and value

The results identified a mediating role for attribution and value. This is consistent with previous partial studies where the relationship between control, value and achievement emotions is largely in line with the predictions of the control-value theory of achievement emotions theory (Pekrun, 2006; Pekrun and Perry, 2014). It is also consistent with previous empirical studies consistent with previous empirical research that autonomy supports positive predictive evaluations of control and value in other academic contexts (Ng et al., 2016; Wang et al., 2017). For example, in a sample of university students who attended tennis courses, control and value appraisal mediated a positive indirect effect of teacher autonomy support on enjoyment, and a negative indirect effect on boredom (Simonton et al., 2021). Similarly, autonomy support provided by PE teachers was a positive predictor of PE-related cognitive evaluations, and control value evaluations were a significant predictor of PE-related achievement mood (Zimmermann et al., 2021). In a sample of secondary school students, students’ intrinsic value in mathematics disciplines, as well as their self-efficacy, mediated the relationship between teacher autonomy support and boredom (Wang et al., 2017). In a sample of university students, the positive relationship between intrinsic value and enjoyment was empirically supported (Simonton and Garn, 2020). Control is positively related to enjoyment, and achievement value, internal value and utility value are positively related to enjoyment (Frenzel et al., 2007a; Pekrun et al., 2011; Hall et al., 2016; Putwain et al., 2021), with low intrinsic value amplifying the relationship between control and enjoyment, i.e., higher values at low control show protective effects at high levels of control and diminish at high levels of control (Hall et al., 2016; Putwain et al., 2021; Zimmermann et al., 2021). When attributions for performance and success tasks are specifically addressed in a learning environment, control may be more predictive of emotional experience (Perry et al., 2001; Pekrun and Perry, 2014). The present study, based primarily on the control-value theory of achievement emotions, confirms the role of attribution and value as mediators between autonomy support and music enjoyment in the context of online music.

According to the results of this study, grades as the control variable is not associated with attributions. In the field of music education, Schmidt (1995) revealed that there is no significant association with attributions and grades when exploring secondary school choral students’ perceived attributions of success to teacher feedback, which corroborates the results of this study. The results also show that the grade is not associated with attainment value and intrinsic value among the values. The findings were supported by studies conducted in the subjects of mathematics, German and English (Arens et al., 2019). Besides, the results of this study found no association between grades and music enjoyment. This is consistent with previous research that focuses on the students’ enjoyment of physical activity, the research showing that there was no difference in the level of enjoyment of participating between the different grades (McMullan et al., 2012). Nevertheless, the results of the study showed a positive association between grades and utility value. Since utility value refers to the extent to which a task fits into an individual’s future plans (Wigfield, 1994). Eccles (2005) explains that utility values can reflect that the activity relates to some important goals held by the person, such as obtaining a certain occupation. Therefore, such results may be due to the fact that the participants in this study are getting closer to their graduation year. The issues of employment or continuing education that are faced near graduation may make students focus more on utility values.

### Implications

This study is one of the few studies in the post-COVID-19 era to examine the association between teachers’ autonomy support strategies and students’ enjoyment of music when taking music courses online for undergraduates who major in music. The study constructs the model by using appraisal as a mediator, and the indirect effects show that value is more relevant than attribution, which explains the role of appraisal as a mediator in detail. This study validates the applicability of the control-value theory of achievement emotions in the context of online music learning during the post-COVID-19 in China, extends the antecedents and mechanisms of action of online music enjoyment emotions, and fills a research gap in online music education.

### Limitations and future study

There are several limitations to this study. Firstly, this study is a cross-sectional survey study and cannot explore the causal relationships between variables. This is one of the main concerns of this study for future research. Secondly, the data was obtained from a self-report questionnaire and although the methodology of this study is not biased, there may be some bias in the respondents’ self-report. In the future, combining self-feedback with other objective records could be considered to improve validity through triangulation (McMurray et al., 2004). Thirdly, the participants in this study were drawn from only one university in the middle of China, and the findings of this study should be extended with caution to a wider range of regions, cultural backgrounds, professions, academic segments or groups (e.g., older people). More groups of students could be involved in the future. Lastly, the sample size of this study could be further increased to enhance the external validity of the study, and future studies could be extended to more regions to explore the association between autonomous support and music enjoyment among music majors both in the forms of online and offline education.

## Conclusion

Based on the context of the popularity of online education in the post-COVID-19 era, this study explored the association between teachers’ autonomy support and students’ music enjoyment in online music learning among undergraduates who major in music. The findings found that Autonomy support positively predicted Music enjoyment, mediated by attribution and value.

## Data availability statement

The raw data supporting the conclusions of this article will be made available by the authors, without undue reservation.

## Ethics statement

The studies involving human participants were reviewed and approved by The Ethics Committee of The Education University of Hong Kong. The patients/participants provided their written informed consent to participate in this study.

## Author contributions

YaZ, YuZ, YL, and XY: conceptualization and methodology. YaZ, YuZ, and YL: formal analysis and investigation and writing—original draft preparation. DT and XY: writing—review and editing, resources, and supervision. All authors contributed to the article and approved the submitted version.

## Conflict of interest

The authors declare that the research was conducted in the absence of any commercial or financial relationships that could be construed as a potential conflict of interest.

## Publisher’s note

All claims expressed in this article are solely those of the authors and do not necessarily represent those of their affiliated organizations, or those of the publisher, the editors and the reviewers. Any product that may be evaluated in this article, or claim that may be made by its manufacturer, is not guaranteed or endorsed by the publisher.

## Footnotes

^1^ http://www.afhayes.com
